# VATS Pleurectomy: A Successful Way to Treat Pneumothorax Recurrence after Blebs Resection

**DOI:** 10.4274/balkanmedj.galenos.2020.2020.4.33

**Published:** 2020-08-11

**Authors:** Marko Bašković, Josip Pejić, Ljudevit Sović, Ante Čizmić, Mirko Žganjer

**Affiliations:** 1 Department of Pediatric Surgery, Children’s Hospital Zagreb, Zagreb, Croatia; 2 Department of Thoracic Surgery, Clinical Hospital Dubrava, Zagreb, Croatia

To the Editor,

A pneumothorax is defined as an abnormal collection of air in the pleural cavity that lies between the visceral and parietal pleura. Spontaneous pneumothorax occurs in the absence of any identified trauma. It is subdivided into primary and secondary types. The annual incidence of primary spontaneous pneumothorax in the general population is estimated to be 5-10 cases per 100,000 population. The peak incidence occurs between the age of 16 and 24 years (1,2).

We present a case of a 16-year-old boy manifested with right-sided spontaneous pneumothorax on several occasions. The boy was actively involved in sports and had practically never been ill before the sudden spontaneous pneumothorax occurred. He did not take any medication and had no allergies. His right chest for drained for a total of three times. At each arrival, pneumothorax manifested with abrupt pain, and each time, the diagnosis was confirmed by an X-ray. After a third spontaneous pneumothorax, a computed tomography scan of the chest was done. The scan showed small apical blebs. The trachea was neatly positioned, neatly branching. The right main bronchus seemed wider than the left. The vascular structures were of a neat appearance. The heart was neatly positioned, with a morphologically neat appearance. Surgical treatment was initiated. After the right lung collapsed, an incision was made in the third intercostal space to the right in the anterior axillary line. An angle camera was installed, and the trocar was placed in the seventh intercostal space in the median axillary line. The apical blebs changes were identified using a linear endostapler, and a resection was performed (Figure 1), followed by a partial parietal pleurectomy (Figure 2). Negative pressure drainage was set. In prophylaxis, the boy received cefazolin. No relapse was reported after a one-year follow-up. Written informed consent was obtained from the patient.

Superficial alveoli can form subpleural blebs that rupture directly into the pleural space. These blebs are usually found in the lung apices, presumably due to the selective ventilation and higher transpulmonary pressures seen in the upper lobes (3). When pneumothorax occurs, the free passage of air into the pleural space allows an equalization of the intrapleural and atmospheric pressures, predisposing to partial lung collapse. A surgery for pneumothorax consists of stapling ruptured blebs and resection of the abnormal lung tissue. The commonly used approaches are video-assisted thoracoscopic surgery (VATS), mini-thoracotomy, and conventional thoracotomy. We usually use VATS, which provides an adequate exposure for a resection or stapling and an opportunity for pleurectomy, abrasion, or chemical pleurodesis. The morbidity of VATS is lower than in the conventional or mini-thoracotomy and the recurrence rate is approximately 5%; however, an open thoracotomy and pleurectomy have the lowest recurrence rate (4). Given that in our previous cases, after repeated tube drainage, no recurrences were reported, we did not opt for VATS immediately after the first recurrence. Based on the literature, in future, we will be guided by an algorithm to initiate VATS after the first recurrence of pneumothorax. In accordance with the research of other authors, we have decided to use VATS pleurectomy as it has been reported to have excellent results when compared with other techniques (5). The patient has given informed consent for publication in this journal.

## Figures and Tables

**Figure 1:**
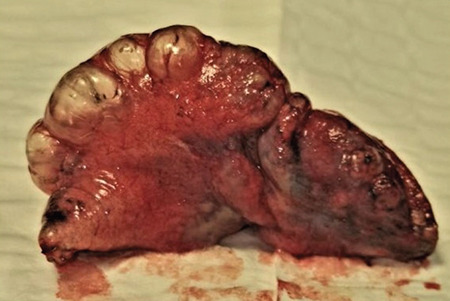
Resected apical portion of lung with blebs.

**Figure 2:**
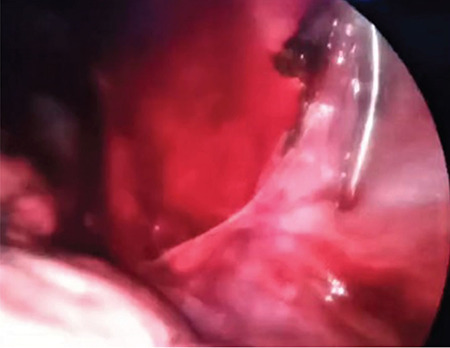
Partial parietal pleurectomy.
